# Comparative Serum Challenges Show Divergent Patterns of Gene Expression and Open Chromatin in Human and Chimpanzee

**DOI:** 10.1093/gbe/evy041

**Published:** 2018-03-05

**Authors:** Jason Pizzollo, William J Nielsen, Yoichiro Shibata, Alexias Safi, Gregory E Crawford, Gregory A Wray, Courtney C Babbitt

**Affiliations:** 1 Molecular and Cellular Biology Graduate Program, University of Massachusetts Amherst; 2 Department of Biology, University of Massachusetts Amherst; 3 Department of Biology, Duke University; 4 Division of Medical Genetics, Department of Pediatrics, Duke University; 5 Center for Genomic and Computational Biology, Duke University; 6 Department of Evolutionary Anthropology, Duke University

**Keywords:** human evolution, late-onset disease, fibroblast, wound healing, epithelial cancer

## Abstract

Humans experience higher rates of age-associated diseases than our closest living evolutionary relatives, chimpanzees. Environmental factors can explain many of these increases in disease risk, but species-specific genetic changes can also play a role. Alleles that confer increased disease susceptibility later in life can persist in a population in the absence of selective pressure if those changes confer positive adaptation early in life. One age-associated disease that disproportionately affects humans compared with chimpanzees is epithelial cancer. Here, we explored genetic differences between humans and chimpanzees in a well-defined experimental assay that mimics gene expression changes that happen during cancer progression: A fibroblast serum challenge. We used this assay with fibroblasts isolated from humans and chimpanzees to explore species-specific differences in gene expression and chromatin state with RNA-Seq and DNase-Seq. Our data reveal that human fibroblasts increase expression of genes associated with wound healing and cancer pathways; in contrast, chimpanzee gene expression changes are not concentrated around particular functional categories. Chromatin accessibility dramatically increases in human fibroblasts, yet decreases in chimpanzee cells during the serum response. Many regions of opening and closing chromatin are in close proximity to genes encoding transcription factors or genes involved in wound healing processes, further supporting the link between changes in activity of regulatory elements and changes in gene expression. Together, these expression and open chromatin data show that humans and chimpanzees have dramatically different responses to the same physiological stressor, and how a core physiological process can evolve quickly over relatively short evolutionary time scales.

## Introduction

Deleterious genetic changes affecting traits that manifest later in life tend to accumulate in long-lived species. Evolutionary theory of aging explains that selective forces are weaker on traits that manifest later in life compared with those that affect survival or fecundity earlier in life (Hamilton 1966; Medawar 1952; Williams 1957). In the absence of purifying selection, late-onset disease alleles can persist or accumulate in a population. Deleterious mutations affecting late-life traits can, however, experience positive selection if they confer positively adaptive changes earlier in life (Carter and Nguyen 2011; Crespi and Summers 2006; Williams 1957). This antagonistic pleiotropic theory of aging may explain why humans, who have long life spans compared with other nonhuman primates, experience higher rates of diseases that manifest later in life compared with our closest living evolutionary relatives (Crespi 2010; Finch 2010).

One of the most prominent diseases of aging affecting the human population is epithelial cancer. In the United States, 86% of cancers are diagnosed in people over 50 years of age (American Cancer Society 2016). There is also a striking difference in the frequency of epithelial cancer in humans compared with chimpanzees (Varki and Varki 2015). In modern human populations, epithelial cancers cause up to 20% of deaths, but in our nearest living relatives, chimpanzees, rates of epithelial cancers are up to 10-fold lower (American Cancer Society 2016; Beniashvili 1989; Hedlund et al. 2007; McClure 1973; Parker et al. 1997; Schmidt 1975; Scott 1992; Seibold and Wolf 1973). It is quite clear that environmental, lifestyle, and dietary factors drive cancer risk (Wu et al. 2016), but genomic differences in humans as compared with other primate species could also play a role. Previous studies suggest that genetic changes in genes associated with cancer are under positive selection in humans and can increase aspects of fitness early in life (Crespi and Summers 2006). While positive selection in regulatory regions does not strictly inform expression differences between species on a gene-by-gene basis, there is a stronger correlation at the level of biological process ontology function (Babbitt et al. 2017), and the differentially expressed genes measured there had an enrichment with cancer-related genes. A similar pattern was found by Nielsen et al. (2005) for genes showing evidence of positive selection in coding regions. Although cancer-related genes were identified among the top 50 genes with signs of positive selection, categorical enrichment for cancer genes within that set was not tested. Increased cancer susceptibility, therefore, may be a trait that has come as a tradeoff as biological processes evolved in humans. Because of the close evolutionary relationship between humans and chimpanzees, understanding genetic differences that contribute to disease phenotypes such as cancer susceptibility can assist in understanding important patterns of functional genetic changes that occurred relatively recently during human evolution.

In this study, we harnessed the power of a well-established experimental assay that models cancer gene expression patterns, allowing us to test the responses of human and chimpanzee cells. When grown in culture, fibroblasts exposed to serum undergo a coordinated pattern of gene expression changes that mimics the wound healing response (Iyer et al. 1999). Tumors have been likened to wounds that do not heal (Dvorak 1986), and these changes in challenged fibroblast gene expression were then subsequently found to strongly correlate with gene expression data from epithelial cancer tissue (Chang et al. 2004). Chang et al. (2005) identified a set of core serum response (CSR) genes that are up- or downregulated independent of the cell-cycle, and the CSR gene expression profile predicts a greater risk for metastasis and death for breast, lung, and stomach carcinomas (Chang et al. 2005). Because humans and chimpanzees have significantly different cancer rates, we hypothesized that the serum response would be significantly different between species.

In order to identify the genetic differences that may drive these important differences in disease phenotypes, we investigated global patterns of gene expression in serum-challenged fibroblasts from humans and chimpanzees using RNA-Seq. All of the genes tested in our assay were analyzed only if there were orthologous genes in both species (Blekhman et al. 2010). To measure dynamic changes in the chromatin landscape, we also sequenced open chromatin using DNase-Seq (Boyle 2008; Song and Crawford 2010) from the same cell population. As a way to investigate biological implications of changes in gene expression and chromatin accessibility, we test for categorical enrichment within gene ontologies, KEGG pathways, and predefined gene sets that are based on knowledge about biological functions. This approach allows us to quantify enrichments based on expression differences and use statistical methods to identify significant changes within gene categories as compared with the background set of all genes measured in this study (Huang et al. 2009b; Subramanian et al. 2005). In our analysis, we found that human fibroblasts undergo distinct physiological changes in response to a serum challenge, including activation of genes involved in homeostasis and cell death. Chimpanzee fibroblasts, however, have a much less focused response, where many genes show differential expression without significant gene ontology enrichment. We also see that serum challenge elicits a general increase in chromatin accessibility in human cells and decreased accessibility in chimpanzee cells. Thus, by using this serum challenge assay in a comparative way, we have identified pathways that differ between species and broad differences in the activation state of cells in response to serum-induced cell stress. This study shows that by using a comparative genomic approach in a model of wound healing and epithelial cancer, we can gain valuable insights into how recent genetic adaptations contribute to differential disease phenotypes between closely related species.

## Materials and Methods

### Serum Challenge

**Fig. 1.**
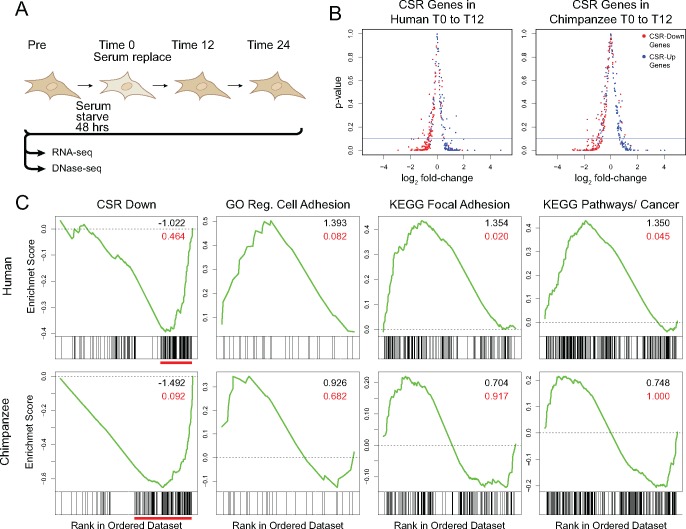
—Characterization of the serum response in human and chimpanzee fibroblasts. (*A*) Overview of experimental procedure. (*B*) Log_2_ fold-change in gene expression versus *P*-value of CSR genes between T0 and T12. Positive log_2_ fold-change indicates higher level of gene expression at T12. Solid blue line indicates *P*-value 0.1. Points are CSR downregulated (red) and upregulated (blue) genes. (*C*) Plots of enrichment scores and distribution of a priori gene sets within the expression set, rank-ordered by differential expression between T0 and T12. Red bars below plots indicate clusters of downregulated CSR genes at the bottom of the ranked list. Inset values are normalized enrichment score (black), and false discovery rate (red).

Fibroblast cell lines were obtained from the Coriell Institute for Medical Research (Camden, NJ) from four male humans and chimpanzees (*Pan troglodytes*; supplementary table S1, Supplementary Material online). Our cell culture methods approximately followed those of Chang et al. (2004) and Iyer et al. (1999). Briefly, we seeded cells in media with FBS (Hyclone defined FBS (-)HI, Fisher) at ∼50% confluency and grew overnight. At 60% confluency one set of plates were set aside and processed as described below (fig. 1*A*, “Pre-challenge” time point). The remainder of the cells were then incubated in starvation media (0.1% FBS) for 48 h, after which the growth media was replaced. Collections were then done at time 0, 12 h, and 24 h. All cells for the RNA-Seq and DNase-Seq experiments were from the same batch and collected at the same time. For the RNA-Seq assays, cells were rinsed with Qiazol (Qiagen) and vortexed. The RNA was isolated using an RNeasy kit (Qiagen), with a DNaseI treatment. For the DNase-Seq assays, ∼20 million cells were spun down and slowly frozen in freezing media (Gibco) to −80 °C.

### RNA-Seq Library Preparation

NGS libraries were prepared using the NEB RNA-Seq library kit for Illumina, and sequenced on a HiSeq at Duke University Genomics Core. Sequences were mapped to the species-specific genome (hg19 and panTro3) using Tophat v.1.4.1 (Trapnell et al. 2009). Counts per gene were determined using HT-Seq (Anders et al. 2015) for genes with clear orthologs in human and chimpanzee (Blekhman et al. 2010). The data were normalized using edgeR (Robinson et al. 2010) with a GLM for multifactor experiments (McCarthy et al. 2012), so that all time point expression was normalized under one model, unless specific time points are mentioned. 199 clones representing 165 genes that were previously identified as being “cell cycle” genes that change through the cell cycle regardless of the serum challenge were removed (Chang et al. 2004; Whitfield et al. 2002).

### DNase-Seq Library Preparation

Library preparation was performed as in Song and Crawford (2010), and sequencing was performed on a HiSeq at Duke University Genomics Core. Sequences were trimmed to 20mer lengths and barcodes removed. In order to compare DHS sites between species, we Bowtie-mapped (Langmead et al. 2009) reads to appropriate genomes and brought chimpanzee coordinates to human space using liftOver (Hinrichs et al. 2006). We called peaks with MACS (Zhang et al. 2008) using a lower *P*-value threshold of 1e-5 and found an average of ∼150,000 peaks in human samples, and ∼116,000 peaks in chimpanzee samples. We counted sites as active if there was a DHS signal in at least 1 replicate. To compare chromatin DNaseI sensitivity at corresponding locations between samples, we defined windows by intersecting DHS sites across all species using BEDTools (Quinlan and Hall 2010). Our approach to defining DHS sites between replicates and between species by using shared “windows” is also graphically explained in the supplementary material (supplementary fig. S1, Supplementary Material online). This gave 379,723 windows between human and chimpanzee samples. We filtered windows to exclude those <50 bp or >2,000 bp, which gave 264,091 sites. The windows from our study include DHS sites that are shared between species, and those that are species-specific. 125,411 sites were shared, 95,983 were human-specific, and only 42,697 were chimpanzee-specific. In any given sample newly defined windows may cover zero or multiple DHS sites. To assign values to each new set of coordinates in each sample, we chose the DHS site with the lowest *P*-value as representative of the activity within each window.

### DHS Window Overlap with ENCODE Data

Data were downloaded from ENCODE that were generated in DNase-Seq experiments in human fibroblasts, cancer cell lines, hepatocytes, pancreas, or cerebellum tissues (supplementary table S2, Supplementary Material online). Additionally, DNase-Seq data from human fibroblasts and LCL cells (Shibata et al. 2012) were used in our fibroblast comparison. Subsets of our DHS sites were selected to test for overlap with external data. Windows were first screened for size and only those between 50 bp and 2 kb were selected. Additionally, a set of “human windows” was selected by removing DHS sites from the 50 bp–2 kb set that did not show any DHS signal at any time point in any human sample. Overlap between our DHS sites and external data was measured using BEDTools (Quinlan and Hall 2010). The exact command used to test for overlap was: Bedtools intersect -u -a OurWindows -b ENCODE_XX_Windows > XX_overlap.

### Gene Set Enrichment Analysis

We tested for enrichment of 12 gene sets downloaded from the Molecular Signatures Database (MSigDB) using the Gene Set Enrichment Analysis (GSEA) desktop graphical user interface (Subramanian et al. 2005). GSEA tests if a predefined set of genes has a statistically significant association with one of two biological states (time points in our assay). We used this analysis to test for enrichment of GO and KEGG gene sets associated with cancer pathways, wound healing, cell adhesion, and for CSR genes. Data in the form of raw read counts were input to test for enrichment between time points during the first 12 or 24 h of the serum response. A rank-ordered list of genes that are statistically different between time points is created, and the positions of a predefined set of genes are determined within this list. An enrichment score is calculated based on a running-sum statistic that increases when a gene is present in the rank-ordered list and decreases when it is not.

### Estimation of Differential Expression

We used edgeR (McCarthy et al. 2012; Robinson et al. 2010) to perform differential expression analysis on RNA-seq and DNase-Seq data sets. Briefly, edgeR fits read counts to a negative binomial generalized linear model and performs a likelihood ratio test to identify differences between groups. We performed this analysis between time points within species to characterize genes that change expression and DHS sites that change activity during the serum response. To characterize between species differences, we performed the analysis at each time point between human and chimpanzee.

### DAVID Enrichment Analysis

We performed differential expression analysis between human and chimpanzee at each of four time points in our experiment. Genes that were significantly (FDR < 0.1) upregulated in each species were selected for enrichment analysis. We sought to investigate enrichments within differentially expressed genes in comparison to a background set of genes expressed in fibroblasts. Thus, we used the Database for Annotation, Visualization and Integrated Discovery DAVID (Huang et al. 2009a, 2009b), which tests for enrichment in the context of a user-defined background. DE gene IDs were supplied to DAVID and the set of all genes active in human and chimpanzee fibroblasts was used as the background. We tested for enrichment using GO biological process annotations for our DE genes and molecular function of subsets of differentially expressed genes within particular biological process categories. For analysis of human enrichments, categories with *P*-values < 0.1 were characterized, whereas for chimpanzee enrichments, we explored categories with *P*-values < 0.25. This less stringent threshold was used for chimpanzee due to the low numbers of categories with significant enrichment values.

### Species-Specific and Serum-Response-Specific Enrichments

In order to determine which categories were enriched at every time point for a particular species, we performed enrichment analysis using DAVID (Huang et al. 2009a, 2009b). Category lists were read into R and were intersected to identify categories that were present in all time points for either human or chimpanzee. To identify BP categories that were enriched during the serum response but not before, for both human and chimpanzee, we read category lists into R, obtained union sets for the Pre and 0 h time points (early set), and then found the set differences between the 12 or 24 h enriched categories and the early set.

### Analysis of Positive Selection in Genes and Promoters

We tested for signs of positive selection in protein coding regions of genes by comparing rates of nonsynonymous and synonymous substitutions (d*N*/d*S*). Data were collected from Ensembl for 12,865 human protein-coding genes and their homologs in chimpanzee (Zerbino et al. 2018). We also looked at d*N*/d*S* for 973 genes that are part of relevant cancer-related categories (Core Serum Response, GO Cell Matrix Adhesion, GO Extracellular Matrix, GO Regulation of Cell Adhesion, GO Response to Wounding, GO Wound Healing, KEGG Basal Cell Carcinoma, KEGG, Cell Adhesion Molecules, KEGG Focal Adhesion, KEGG Pathways in Cancer, Mishra Carcinoma Associated Fibroblast UP). Gene lists were collected from the MSigDB (Subramanian et al. 2005).

Additionally, we looked for signs of positive selection in human promoter sequences (5 kb regions upstream of transcription start sites) using code from Haygood et al. (2007) available on GitHub (https://github.com/ofedrigo/TestForPositiveSelection) for 5,137 genes with clear orthology in chimpanzee and rhesus macaque (*Macaca mulatta*), which we use as an outgroup in this analysis. Briefly, this code runs using HyPhy software (Pond et al. 2005), and calculates nucleotide substitution rates in promoter sequences and compares this to neutral substitution rates in nearby intronic regions (first intron of a genes were excluded). *P*-values were used to identify promoters with significantly higher rates of substitution on the human branch.

### Identifying Putative Transcription Factor Binding Sites Using JASPAR

In order to identify known transcription factor binding motifs that are contained within our DHS sites, we collected hg19 sequences corresponding to our DHS coordinates and scanned these sequences for known motifs that are described in the JASPAR database (Sandelin et al. 2004). We searched for all *Homo sapiens* motifs on both + and – strands and specified a minimum score of 100% using R packages TFBSTools (Tan and Lenhard 2016) and JASPAR2016 (Mathelier et al. 2016).

### Fuzzy Clustering Analysis

We performed soft clustering of DHS data using mFuzz (Futschik and Carlisle 2005), using standardized expression sets prepared from log_2_ values of mean DHS activity among replicates for shared or species-specific DHS sites. The fuzzifier for each expression set was selected with the mestimate function. Minimum centroid distances were calculated for a range of cluster numbers using Dmin, and an optimal number of clusters was chosen to select the lowest centroid distance with the lowest number of clusters. For clusters that represent increases or decreases in DHS activity during the serum response, we selected DHS sites within each cluster with a minimum membership value of 0.6, and identified genes closest to each DHS site using UCSC (Karolchik et al. 2004) gene coordinates for genes with clear orthologs in human and chimpanzee (Blekhman et al. 2010). We tested for biological process category enrichment from these genes with GOrilla (Eden et al. 2009), which allows testing large data sets against a background. The set of all genes active in human and chimpanzee fibroblasts was used as the background.

## Results

### The Serum Response in Both Human and Chimpanzee Fibroblasts Is Similar to the Established CSR

CSR genes are upregulated or downregulated upon serum challenge; Chang et al. (2004) used microarrays to identify a set of 512 genes they defined as part of the CSR. To define this response they used the assay described above, where fibroblasts are grown in vitro, are starved of serum for 48 h, and are subsequently re-exposed to normal levels of serum in the culture medium. The 512 CSR genes are now part of a curated data set in the MSigDB (Subramanian et al. 2005), which includes 212 upregulated and 209 downregulated genes. As a first analysis of our data, we wanted to see a replication of this response in our cells and gene expression platform. Although we compare data compared across platforms, we expect to find similar patterns of expression. The same sets of genes were tested in both studies, and previous comparisons of RNA-Seq and microarray data show significant correlation of expression profiles between platforms (t Hoen et al. 2008; Zhao et al. 2014).

We measured the serum response in fibroblasts from humans (four biological replicates) and chimpanzees (three biological replicates), examining gene expression by RNA-Seq at four time points (fig. 1*A*). Of 421 CSR genes, 324 overlapped genes in our results. Part of the reason we test a subset of these genes is because throughout our analysis we only compared genes that have clear orthologs in both humans and chimpanzees. In both species, the majority of CSR genes increase or decrease expression levels as expected based on the work of Chang et al. (2004) (fig. 1*B* and supplementary fig. S2, Supplementary Material online). In human fibroblasts, 84% and 89% of CSR-Up genes are upregulated at 12 and 24 h, respectively, and in chimpanzee fibroblasts, 73% and 87% are upregulated at 12 and 24 h. Similarly, 88% of CSR-Down genes are downregulated in human fibroblasts, and in chimpanzee fibroblasts, 90% and 84% are downregulated at 12 and 24 h. Of the genes that increase or decrease expression as expected, there are comparable numbers of significant (FDR ≤ 10%) differences over time in both species. These changes in expression through the time-course of the assay are much as expected for a core biological process. Beyond these CSR genes, however, we see between-species differences in functional categories of genes that are involved in important aspects of physiology.

### Wound Healing and Cancer Pathway Genes Increase Expression in Human, but Not Chimpanzee, Fibroblasts during the Serum Response

In order to explore the biology of the serum response across species, we tested for gene ontology enrichment categories between time points that characterize the serum response pathways using GSEA (supplementary table S3, Supplementary Material online; Subramanian et al. 2005). Although CSR upregulated genes are significantly (FDR < 0.1) enriched at 12 h in both human and chimpanzee, the enrichment for CSR downregulated genes is only significant in chimpanzee (fig. 1*C* and supplementary table S3, Supplementary Material online). In human fibroblasts, however, GO cell adhesion, Kyoto Encyclopedia of Genes and Genomes (KEGG) focal adhesion, and KEGG cancer pathway genes are significantly enriched at the 12-h time point in human, but not in chimpanzee. These results suggest that although the CSR is similar between species, there are important wound healing and cancer-related pathways that are upregulated in human fibroblasts but not in chimpanzee.

### Human Fibroblasts Have a Coordinated Homeostasis and Cell Signaling Response to Serum

In order to understand how human and chimpanzee fibroblasts respond to serum on a gene-by-gene level, we next performed differential expression analysis between species at each time point (McCarthy et al. 2012; Robinson et al. 2010). This analysis shows that there are more DE genes (FDR ≤ 10%) with higher expression levels in chimpanzee than human in every comparison, with an average of about twice as many DE genes with higher expression in chimpanzee (table 1).

**Table 1:** Differential Gene Expression between Species at Each Time Point (FDR 10%)

	Number of DE Genes (FDR 10%)			
	Pre	T0	T12	T24
Higher in human	910	925	983	856
Higher in chimpanzee	1,460	2,203	1,431	2,350
Total DE genes	2,370	3,128	2,414	3,206
Ratio chimpanzee DE:human DE	1.60	2.38	1.46	2.75

To investigate processes that are unique to the serum response, we measured enrichment for BP categories (Huang et al. 2009a, 2009b) from differentially upregulated genes within each species, and identified the categories that were enriched during the serum response (T12 and T24), but not at earlier time points (Pre and T0). At 12 and 24 h in human fibroblasts, there were 23 and 27 BP categories, respectively, with *P*-values ≤ 0.05 (supplementary table S4, Supplementary Material online). Most of these processes describe homeostasis or cell signaling and protein modification processes. As well, several processes related to cell death, development or morphogenesis, and response to stimuli are enriched. At 12 and 24 h in chimpanzee fibroblasts, there were only 2 and 37 categories, respectively, with *P*-values ≤ 0.05. The enriched processes at 12 h are cell proliferation and proximal/distal pattern formation, and at 24 h the majority of processes relate to cell cycle and metabolic processes. The processes identified in humans describe a broad response to stress and stimuli and show that fibroblasts initiate mechanisms to cope with a changing external environment due to the presence of serum. On the other hand, gene expression in chimpanzee fibroblasts is not enriched for any one aspect of physiology related to the serum response; instead it is much more diffuse over biological processes. These results suggest that the chimpanzee cells are reacting in a less coordinated way to regulate cell state during times of stress.

### Genes with Higher Expression in Human Fibroblasts Are Enriched for Development, Adhesion, and Angiogenesis Categories

**Fig. 2.**
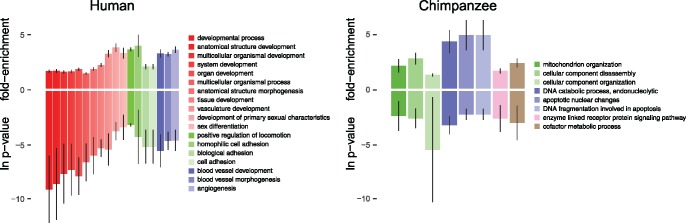
—Common GO terms across time points. GO BP categories significantly higher in human and chimpanzee at all time points. Bars represent mean values across 4 time points for ln *P*-value (below axis) and fold-enrichment (above axis). Colors group similar biological processes and vertical lines represent 1 standard deviation from the mean.

We were interested if differences in gene expression could inform about fundamental differences in physiology between the two species. To explore this, we looked for BP categories that were enriched at all time points for human or for chimpanzee. These common categories may represent broad biological processes that are uniquely elevated in one species. Genes that were more highly expressed in human at Pre, T0, T12, and T24 shared enrichment (*P* ≤ 0.1) for 18 categories across time points (fig. 2). These include 11 human BP categories that represent development, morphogenesis, or differentiation, four categories related to locomotion or adhesion, and three categories related to angiogenesis and blood vessel development.

Molecular function enrichment of the genes in the 11 development categories indicates that transcription factor activity is highly enriched at each time point, with *P*-values ≤ 1.1 × 10^−3^ and at least 3.3-fold enrichment. Transcription factors within this set of 22 genes have normal roles in embryogenesis and angiogenesis during wound healing, but are often aberrantly regulated in cancers (Abate-Shen 2002; Gilkes et al. 2014). Angiogenesis is an essential process in normal wound healing that allows for the delivery of oxygen and nutrients to the site of injury, and plays an important role in the formation of new tissue (Tonnesen et al. 2000). In a similar fashion, development of new vasculature is essential for the continued growth of tumors and metastasis (Yadav et al. 2015). Together, these data show that genes with higher expression in human fibroblasts enrich for critical parts of the wound healing process at all time points.

Using the same between-species differential expression and biological process enrichment data set we looked for processes that were shared in chimpanzee between all time points. Here, we found no common BP categories with significant enrichment *P*-values (*P* ≤ 0.1). However, in order to explore some categories that may be enriched in chimpanzees, we relaxed this requirement to categories with *P*-value ≤ 0.25 and found eight categories that were shared between time points (fig. 2). Similar to our analysis of processes unique to the serum response, the lack of categories that are enriched across time points indicates that elevated gene expression in chimpanzee fibroblasts is distributed across many processes, as opposed to the stronger enrichments we see in the human gene expression data. As an exploratory analysis, we also note a few categories that stand under the less stringent *P*-value threshold applied to chimpanzee enrichments. Here, DNA catabolism and fragmentation, and apoptotic nuclear change categories have a high fold-enrichment, between 2.9 and 6.3, but *P*-values are between 0.02 and 0.2. At the 24-h time point, cellular component organization has a very low *P*-value (4 × 10^−6^; which accounts for the high standard deviation), but does not have a high fold-enrichment. Thus, while the error rates associated with these enrichments increase with the less stringent *P*-value applied to these data, in the absence of highly significant enrichments, we can nonetheless gain insight into possible biological characteristics common to chimpanzee fibroblasts.

### Genes with Signs of Positively Selected Changes in Protein Coding Regions Are Not Enriched among Cancer-Related Genes

While differences in levels of gene expression play an important role in controlling phenotypes (Wray et al. 2003), nucleotide level differences in protein coding genes within relevant disease pathways may also contribute to differential disease susceptibility (Puente et al. 2006). To explore genetic differences between species, we looked at nucleotide level differences in human and chimpanzee orthologs from publicly available genome sequence (Zerbino et al. 2018).

We first looked at rates of nonsynonymous substitution (d*N*) in 973 genes that are important in wound healing and cancer pathways and are part of well-defined gene sets in the MSigDB. We made estimates for each gene by selecting isoforms with the highest d*N* values. The average d*N* values for cancer-related genes and the full set of 12,865 homologues quantified in this study are comparable at 0.008 and 0.0086, respectively indicating that there is no significant enrichment for nonsynonymous substitutions in cancer-related genes (two-sided Fisher's exact test *P*-value = 1).

In order to see if there are signs of positive selection in coding regions of cancer-related genes, we looked at ratios of nonsynonymous to synonymous substitutions (d*N*/d*S*) for all genes tested in our study (supplementary table S5, Supplementary Material online). Only 5.14% (50 of 973) of cancer genes have d*N*/d*S* > 1, whereas this rate is 7.55% (971 of 12,865) for all genes in our study (two-sided Fisher's exact test *P*-value = 0.00851). This lower rate suggests that there is no enrichment for positively selected protein coding changes in cancer-related genes as a whole. Nonetheless, because positively selected changes in individual genes could contribute to differential phenotypes, we looked more closely at which genes have the greatest signals of positive selection.

We looked in the set of cancer-related genes with d*N*/d*S* > 1 and overlapped that with genes that have significantly higher expression in humans. In the gene coding for CXCL6, a ligand for chemotaxis and angiogenesis, d*N* = 0.0167, which is in the 90th percentile of d*N* scores of all 12,865 genes in our study. The reported d*S* value is 0, preventing an exact calculation of d*N*/d*S*, but highlighting the excess nonsynonymous substitution rate. The genes encoding MXI1 and NKX3-1, both tumor suppressors, also have signatures of positive selection where d*N* = 0.0034 and 0.0053, respectively, and d*S* for each = 0. We further looked at genes with the highest d*N*/d*S* ratios to explore biological processes where we see strong signals of positive selection. Here, we see that genes that have roles in spermatogenesis (*DNAL1*, *TMEM225*, *TRIM69*, *TMCO5A*), metabolism (*DHRS12* and *NUDT17*), immune responses (*IL15RA* and *TRAFD1*), or apoptosis (*DFFA*) show the highest signals of positive selection (supplementary table S5, Supplementary Material online). These data align with previous reports that these biological processes contain an excess number of genes with positively selected changes (Bustamante et al. 2005), and it is possible that genes with such changes could contribute to overall differences in disease susceptibility between species. While our data do show that there are some changes in genes that have possible roles in disease susceptibility, the impact of these individual changes on overall disease incidences is not clear. It is possible that there could be some protein coding changes that contribute to disease processes, but there is not a statistical enrichment for positively selected changes in cancer-related genes as a whole.

### Genes Upregulated in Human Fibroblasts Show Signatures of Positive Selection

Global analysis of *cis*-regulatory sequences within promoter regions has revealed evidence of positive selection in humans for genes involved in neural development and glucose metabolism (Haygood et al. 2007). Because we see particular biological processes enriched in humans at all time points and specifically during the serum response, we wondered if regulatory regions near genes that contribute to these enrichments show evidence of positive selection. In humans, we see that enriched biological processes are important for wound healing and cancer progression, and selection in noncoding regions around these genes could suggest adaptation in the form of changing gene regulation. We compared genes identified by Haygood et al. (2007) that show signs of positive selection in regulatory regions with those that contribute to biological processes that are upregulated in human fibroblasts and found some overlap between the two data sets. These include *MMP8*, a metallopeptidase that contributes to extracellular matrix remodeling, *NAALAD2*, a peptidase that hydrolyses *N-*acetyl-aspartyl glutamate and glutamate and a marker of prostatic carcinomas, and *ACVRL1*, a receptor for TGF-β family of ligands. Outside of those that contribute to enriched processes are genes that are elevated in human fibroblasts at all time points. These include *ALS2CL*, *RGS20*, and *SNX16* involved in cell signaling, *DPT*, which has a role in cellular adhesion, and *PFKFB3* involved in the control of glycolysis. These results suggest that while there are individual genes that are significantly upregulated in human fibroblasts that have signs of positive selection, these are not focused on any one biological process. Selection around these genes, however, does show that there are changes in processes important for fibroblast function, wound healing, and cancer progression. Because chronic wound healing processes are co-opted by developing tumors, adaptation for higher expression within these processes in humans could help explain increased disease susceptibility.

To look more closely at how positive selection may be shaping gene expression in humans, we used methods adapted from Haygood et al. (2007) to test for signs of selection in promoter regions of genes in our study. We used a set of 5,137 genes that have orthologs in human, chimpanzee, and rhesus macaque and compared substitution rates in promoters to intronic sequence, which serves as a measure of neutral substitution rates. Here, we see 3.5% of promoters have signs of positive selection indicating that these events are relatively rare in humans (likelihood ratio test *P*-value ≤ 0.01). To find out where these selection events are happening, we performed an enrichment analysis for GO biological processes. Among the most enriched processes are those related to neural function including anion transport, sensory perception of light stimulus, and visual perception, which is in agreement with the Haygood et al. (2007) findings that neural genes have experienced recent positive selection in proximal regulatory regions (supplementary table S6, Supplementary Material online). Genes contributing to these enrichment include *GLRA1*, which mediates central nervous system postsynaptic inhibition, *FAM161A*, involved in retinal progenitor cell proliferation, *NDP*, involved in retinal vascularization, and *CNGA1*, which is involved in phototransduction. Also included are transcriptional regulators *MAP2K6* that regulates stress induced cell cycle arrest, transcription activation, and apoptosis, and *POU6F2* which is a tumor suppressor involved in nephroblastoma predisposition (Di Renzo et al. 2006). While signs of positive selection do not fully explain differential gene expression in humans, our results here do agree with previous reports that neural-related processes and control of transcription are enriched for signs of positive selection. While we are beginning understand how individual genes can show positively adaptive function and also contribute to disease processes (Crespi and Summers 2006), we understand less about how adaptation and antagonism occur broadly across functional biological process categories. Nonetheless, signs of adaptation around individual genes can offer some insight into how physiological responses change over evolutionary time.

### Human Chromatin Has Significantly More Open Chromatin than Chimpanzee

Layering in a second data set, we examined global changes in chromatin accessibility over the challenge time points using DNase-Seq (Boyle 2008). Regions of open chromatin are susceptible to DNaseI cleavage (Keene et al. 1981) and sites hypersensitive to DNaseI mark many types of regulatory elements (Gross and Garrard 1988). As a preliminary characterization, we looked to see if the locations of our DHS sites have been identified in previous studies that used DNase-Seq, we scanned for the presence of transcription factor binding motifs, and looked at how openness of DHS sites changes relative to proximity to transcription start sites (supplementary text, Supplementary Material online). Together, these characteristics suggest that the DHS sites we identified contain functional elements and help to validate our DNase-Seq data set.

**Fig. 3.**
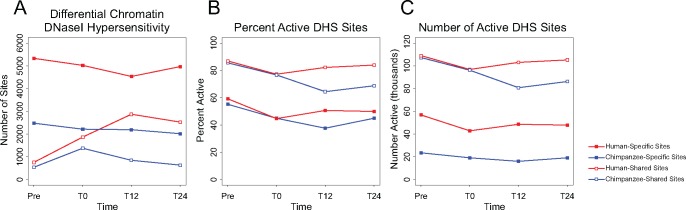
—Differential chromatin accessibility in human and chimpanzee fibroblasts. (*A*) Differential chromatin DNaseI hypersensitivity was determined using a 5% FDR from a likelihood ratio test. (*B*) and (*C*) Active DHS sites are locations in which a DNaseI signal with a *P*-value of at least 1e-5 is present in at least one replicate. Red lines with filled markers represent activity of human-specific DHS sites. Blue lines with filled markers represent activity of chimpanzee-specific DHS sites. Red lines with empty markers represent activity of shared DHS sites in human fibroblasts. Blue lines with empty markers represent activity of shared DHS sites in chimpanzee fibroblasts.

In order to compare the activity of DHS sites between species, we used a 5% FDR to identify significant differences at each time point. There are ∼9,000–10,000 sites with significantly different DHS signals at every time (fig. 3*A*). Importantly, not all DHS sites are present in both human and chimpanzee fibroblasts. To more deeply investigate how chromatin changes during our assay, we identified sites that were shared between species, and those that are species-specific. Many of the significant differences in DHS signal are, appropriately, in species-specific DHS sites. However, of the sites that are shared between species, there are significantly (Fisher's exact test *P*-value ≤ 2.196 × 10^−9^) more sites with higher DNaseI sensitivity in human at all time points. During the serum challenge, chromatin containing DHS sites shared between species increases accessibility in human fibroblasts and decreases in chimpanzee. This, along with the larger number of human-specific sites indicates greater chromatin accessibility in general (Pre and T0), and in response to stress (T12 and T24) in human fibroblasts.

Not all of the DHS sites identified are active at all time points. In terms of percentage of active sites at any given time point, more shared sites are active than species-specific sites (fig. 3*B*). Interestingly, even though humans have twice as many active species-specific sites as chimpanzee (fig. 3*C*), the percentage of these sites that are active through the assay is comparable (fig. 3*B*), suggesting that technical differences in genome annotation are not responsible for higher levels of species-specific DHS sites in human. While the total number of active DHS sites in human fibroblasts remains relatively stable, chimpanzee chromatin shows a decrease in accessibility during the first 12 h of the serum challenge, particularly at shared DHS sites (fig. 3*C*). These data suggest that chimpanzee chromatin responds to the stress of the serum challenge with a general decrease in accessibility.

### DHS Sites That Cluster in Patterns of Opening and Closing Chromatin Reflect Functional Control of Transcription and Adhesion Processes

**Fig. 4.**
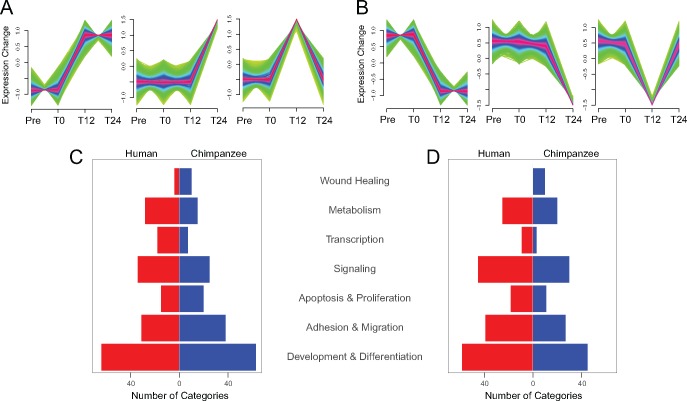
—Fuzzy clustering and ontology enrichments. Clusters represent either an (*A*) increase or (*B*) decrease in chromatin accessibility following serum replacement. (*C*) and (*D*) Common GO terms generated from genes nearest DHS sites belonging to clusters that indicate (*C*) increasing or (*D*) decreasing chromatin accessibility.

In order to explore temporal patterns of chromatin state, we performed fuzzy clustering (Futschik and Carlisle 2005) of the mean -10log_10_*P*-values of DHS sites within each species during the serum challenge. Most clusters have a bimodal shape, which generally describes a site as open or closed at a given time point. Interestingly, the top three cluster shapes with the highest membership values are the same in both human and chimp (supplementary fig. S5, Supplementary Material online). There are particular clusters that are interesting in terms of activity, specifically in response to the serum challenge. These represent DHS sites in which changes in chromatin openness occur: At the beginning of the challenge and persist, at the end of the challenge, or at the beginning of the challenge that revert (fig. 4*A* and *B*). In general, there are multiple DHS sites in proximity to each gene. While there appear to be comparable numbers of DHS sites opening and closing during the serum challenge in chimpanzee, there are ∼4.5× as many (19,974/4,462) DHS sites in human that fit in clusters representing chromatin opening compared with clusters representing chromatin closing (table 2). These 4,462 DHS sites that represent chromatin closing are found in proximity to 3,163 genes. Thus, when DHS sites are closing in human fibroblasts, there is frequently only about one site that closes per gene, whereas in chimpanzee there are ∼2 sites that close per gene. When DHS sites are opening in human there are 2.74 sites per gene, and in chimpanzee, there are 2.25 sites per gene. Not only are there a larger absolute number of sites of opening chromatin in human, these sites are more concentrated around genes than they are in chimpanzee, and the change to a closed state in DHS sites in human is less concentrated around genes than in chimpanzee.

**Table 2:** DHS Sites and Genes Associated with Clusters Representing Opening and Closing Chromatin during the Serum Challenge

	Chromatin Opening		Chromatin Closing	
	Human	Chimpanzee	Human	Chimpanzee
Number DHSs	19,974	13,282	4,462	13,350
Number genes	7,286	5,910	3,163	6,501
ratio DHSs/gene	2.74	2.25	1.41	2.05

As a way to investigate the biological processes that may be controlled by these regions of opening or closing chromatin, we performed gene ontology enrichment analysis (Eden et al. 2009) of genes closest to DHS sites belonging to clusters that represent chromatin opening or closing during the serum challenge. Among the enriched categories, particular processes were common between species. We computationally grouped these processes into representative categories using key terms (fig. 4*C* and *D*). For example, the “Development” category represents biological processes of development, morphogenesis, and differentiation, and the “Growth” category represents processes of growth, proliferation, death, and apoptosis. All processes were enriched with *P*-values < 10^−3^. Grouping these categories shows common themes among the enrichments and shows similar themes to gene expression enrichments. Development categories are among the most prevalent, and similar to gene expression enrichments, transcription factor activity is one of the most highly enriched molecular functions from these genes. In agreement with gene expression enrichments, motility, adhesion, migration, and growth, death, apoptosis, and proliferation are enriched in up- and downregulated clusters. Although there are a small number of categories related to wound healing, it seems this response is more highly enriched in chimpanzee than human.

### Positive Correlations Exist between DHS Sites and Levels of Gene Expression

Next, we wanted to bring our two data sets (RNA-Seq and DNase-Seq) together to examine how DHS activity and distribution compares with expression. Linking gene regulation and gene expression at a whole-genome level is notoriously difficult because regulatory elements can act at large distances from, and independent of orientation to, target genes. In the absence of annotated relationships between regulatory elements and target genes, linking putative regulatory elements and genes based on proximity is the most feasible route to explore these relationships on a genome-wide scale. Boyle (2008) have shown that there is a low correlation when directly comparing expression and the degree of hypersensitivity, but there is a significant difference between DNaseI sensitivity at the TSS of low or no expression compared with genes with moderate or high expression. Additionally, they show that many of the most active DHS sites are in promoters and within the first exon.

To look at relationships between DHS signal and gene expression, we calculated the total DHS signal for all DHS sites closest to all TSSs, and performed a Spearman's correlation test against gene expression values for these genes. In both human and chimpanzee at each time point, Spearman's rho was between 0.24 and 0.25, indicating that there is a weak but positive correlation between DHS activity and gene expression (supplementary fig. S6, Supplementary Material online). There is a stronger correlation, however, between log_2_ fold-change in gene expression between species and the ratio of active DHS sites per gene. At each time point, the Spearman's rho is between 0.327 and 0.487 and *P*-value is < 2.2e-16. These data show that positive relationships exist between DHS activity and gene expression based on proximity of DHS sites and TSSs, but individual metrics describing DHS activity are not strong predictors of gene expression at the closest TSS.

## Discussion

In response to a serum challenge, fibroblasts undergo a defined transcription activation profile that mimics the wound healing response (Chang et al. 2004) and is similar to the expression profile found in tumors (Chang et al. 2005). In our experiments, we found a gene expression profile in both species that mimics the CSR described by Chang et al. (2005). There are differences in the response; however, and some of these may be explained by updated gene models and differences in experimental platforms between the studies, changing from microarrays to RNA-Seq. By using this assay in two closely related species with prominent phenotypic differences in wound healing and cancer rates, we are able to investigate genetic differences during the serum response in this physiologically relevant cell type that have evolved over a relatively short timescale (∼5–7 million years; Chen and Li 2001; Langergraber et al. 2012).

Focusing on gene expression, our RNA-Seq data suggest that the CSR is similar between species; yet, there are important wound healing and cancer-related pathways that are upregulated in human fibroblasts but not in chimpanzee. While chimpanzees have more genes with higher levels of expression than humans, these are unfocused and not enriched for specific biological processes. Humans, on the other hand, have fewer genes with higher levels of expression than chimpanzee, but these are contained within particular biological processes and pathways. Genes encoding transcription factors enrich process of development, morphogenesis, or differentiation in human fibroblasts. Cell adhesion processes are also enriched in humans at all time points. This difference has been identified previously in gene expression and cellular focal adhesion comparisons in human and chimpanzee fibroblasts (Advani et al. 2016). These molecules play critical roles in the function of fibroblasts by mediating cell–cell and cell–matrix interactions and have important roles in cell responses to external stimuli (Calvo et al. 2013; Clayton et al. 1998).

To look at how genetic differences between species could be affecting phenotypes, we looked at how protein coding regions and gene promoters differ between species. We found signs of positive selection in protein coding genes that are part of cancer-related pathways, but no enrichment among those processes. Some genes that are differentially expressed in humans have signs of positive selection and are part of cancer pathways, but a direct relationship between genetic changes and species phenotypes is difficult to make. Likewise, our analysis of positive selection in promoter regions found enrichments for neural-related processes, but these events are rare, only occurring in ∼3.5% of the promoters tested. Here, we found that promoters with signs of positive selection have roles in neural function including visual system development and differentiation, and anion transport. These positively selected changes agree with known differences in species biology, but do not fully explain how selection in upstream regulatory regions contributes to gene expression.

The expression level of any gene is dictated by the activity of regulatory elements that promote or repress transcription. However, the relationship between number, location, and activity of *cis*-regulatory elements and associated genes globally is not clear. Because we know that DHS sites mark regulatory elements, we can still, however, identify differences in availability of putative regulatory elements available to cells at each time point. Our DNase-Seq data show that human cells have higher levels of chromatin accessibility at all time points. The increased chromatin openness is somewhat counterintuitive considering the higher levels of gene expression seen in chimpanzee. One would expect that increased chromatin accessibility in human fibroblasts would result in higher levels of gene expression, but this is not the case. This may indicate that human cells exist in a more poised, or transcription-ready, state than chimpanzee cells. Open chromatin data showing a higher level of chromatin accessibility, and expression data showing significant changes in transcription factor activity, together suggest that human cells maintain a transcription-ready state, which could allow for a faster transcriptional response.

Understanding how these regions of open chromatin might be driving changes in gene expression remains a challenge. Because regulatory elements can act at large distances relative to their target genes, linking gene expression and regulation is difficult. Between our open chromatin and gene expression data sets, we only found weakly positive correlations based on sequence proximity. To identify specific links between regulatory elements and a specific gene expression level will require more targeted experiments, such as luciferase assays.

Comparative approaches to studying genomic differences between species make extensions of our knowledge of biology across species. Although some assumptions are made about functional conservation, genetic differences between species correlate with known differences in species biology. In taking a comparative approach to investigating genomic responses to a well-defined experimental assay in two closely related species, we begin to explore how genetic changes functionally contribute to differences in a core physiological process over a relatively short evolutionary time scale. Here, we see that humans and chimpanzees have very different responses to the same physiological stressor. The human response is generally rapid and robust with focused changes in gene expression and chromatin openness around functional groups of genes important for wound healing. This response may be part of a genetic adaptation that allows for quick mobilization of transcriptional programs to cope with changing extracellular state. The ability to quickly engage robust genetic responses to a wound healing stimulus or respond to other stimuli could have important adaptive function (de Nadal et al. 2011; Lopez-Maury et al. 2008). However, a strong or prolonged wound healing response in the context of a cancerous lesion could be deleterious. While this experimental assay does not describe mechanisms that initiate disease, it does serve as a way to explore how genetic programs that significantly differ between humans and chimpanzees could contribute to increased disease susceptibility in humans.

Striking differences in the rates of epithelial cancers exist between humans and chimpanzees. The lifetime risk for development of cancer depends on the effects of acute and cumulative exposure to environmental factors, but also on genetic defects and predisposition (American Cancer Society 2016; Lichtenstein et al. 2000; Stearns and Medzhitov 2016). Certain environmental factors play a large role in human exposure (American Cancer Society 2016) but not in chimpanzees, while exposure to carcinogens through other environmental factors may be more similar between species (Varki and Varki 2015). While part of the difference in cancer rates between our species is due to these external factors, genetic differences likely play a role as well. Comparative genomics allows for investigation of global differences in gene expression and chromatin responses between humans and chimpanzees. This approach can identify genetic changes that occurred as humans diverged from the most recent human–chimpanzee ancestor, which may be responsible for particular phenotypes (Olson and Varki 2003), and which may be driven by differential gene expression rather than changes in protein coding regions (reviewed in Carroll 2005; Wray et al. 2003). Changes in the activity of regulatory elements can offer a mechanistic explanation for differential expression between species. These changes can occur rapidly over evolutionary time, such as the ∼5–7 mya divergence measured here, possibly driven by positive selection giving rise to new phenotypes. Along with positive adaptations, however, can come side effects that manifest later in life-history as unfavorable phenotypes such as disease susceptibilities. These unintended changes might not be readily visible to selection and may be propagated over evolutionary time. The use of evolutionary comparisons to better understand shifting rates of disease between humans and nonhuman primates can be used as a valuable tool for studying genetic factors that confer uniquely human characteristics and disease susceptibilities.

## Supplementary Material

Supplementary data are available at *Genome Biology and Evolution* online.

## Supplementary Material

Supplementary Data
