# Supplementary material for: Comparative Serum Challenges Show Divergent Patterns of Gene Expression and Open Chromatin in Human and Chimpanzee
Source: Genome Biol Evol. 2018 Mar 5;10(3):826–39. doi: 10.1093/gbe/evy041 (PMC5848805; doi:10.1093/gbe/evy041)
Supplement: Supplementary Data [file evy041_supp.zip › Supplementary_figures.pdf]

Figure S1

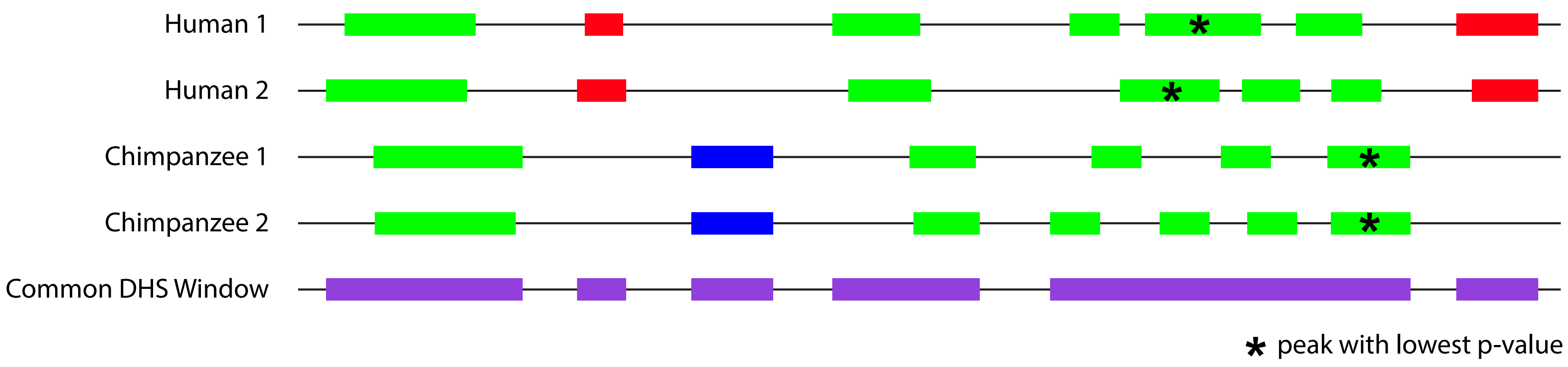

Figure S1. Scheme describing definition of DHS windows. Purple bars display coordinates that encompass overlapping DHS sites from multiple replicates and/ or species. Green bars represent DHS sites shared between species. Red bars represent human-specific DHS sites. Blue bars represent chimpanzee-specific DHS sites. When multiple DHS sites fall within the same window, the lowest p-value of DHS sites (denoted with an asterisk) within the window is used as representative of DHS activity at the merged coordinates.

Figure S2

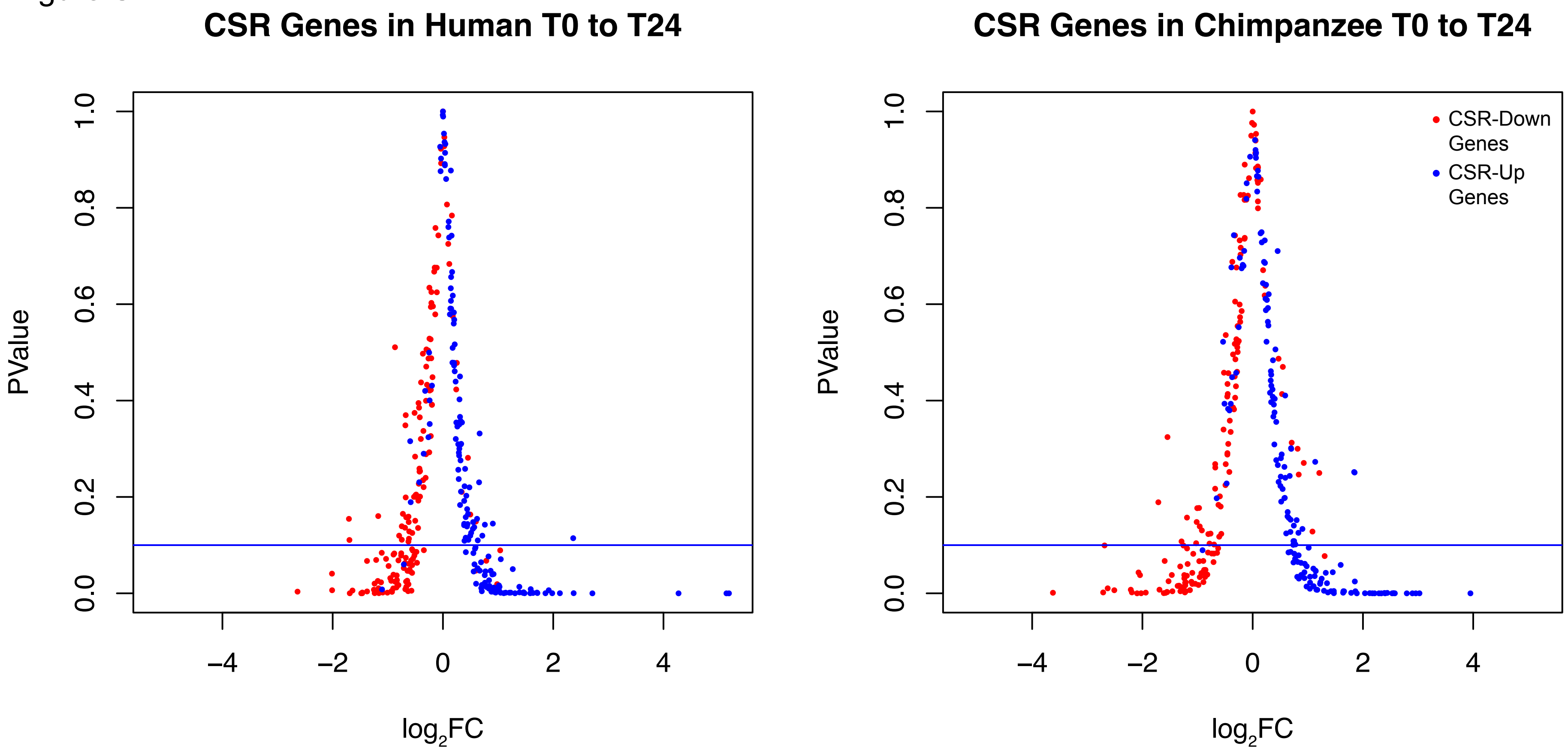

Figure S2. Expression of CSR genes during the first 24 hours of the serum challenge. Log_2 fold-change in gene expression versus p-value of CSR genes between T0 and T24. Positive log_2 fold-change indicates higher level of gene expression at T24. Solid blue line indicates p-value 0.1. Points are CSR down-regulated (red) and up-regulated (blue) genes.

Figure S3

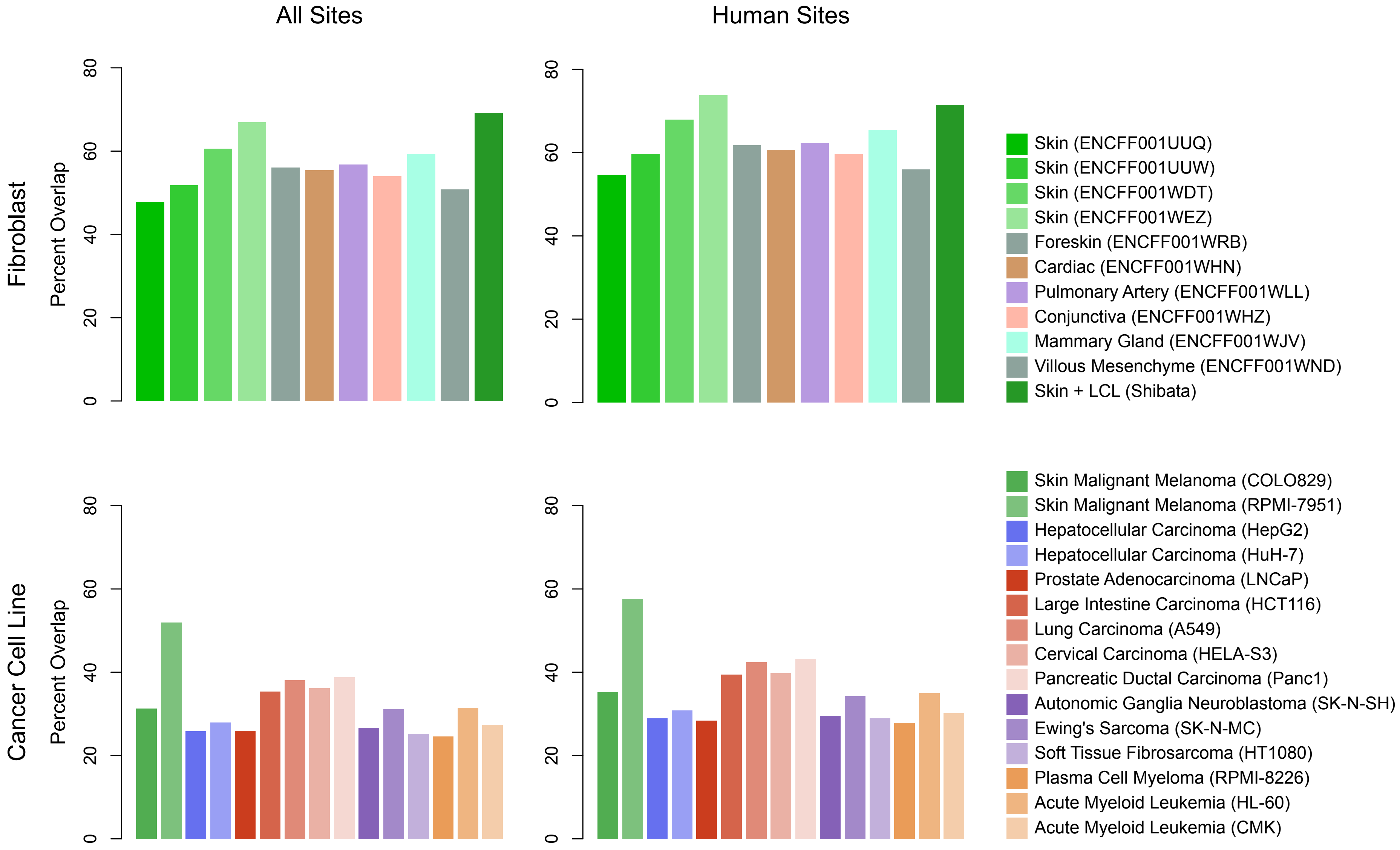

Figure S3. Overlap of DHS sites with previously identified sites. Percent overlap indicates the percent of our DHS sites which overlap by at least 1 bp with the location of DHS sites from ENCODE accessions. All sites include human-specific, chimpanzee-specific, and shard sites. Human sites include human-specific and shared sites. Shibata et al. data include DHS sites identified in skin fibroblasts plus lymphoblastoid cell lines (LCLs).

Figure S4

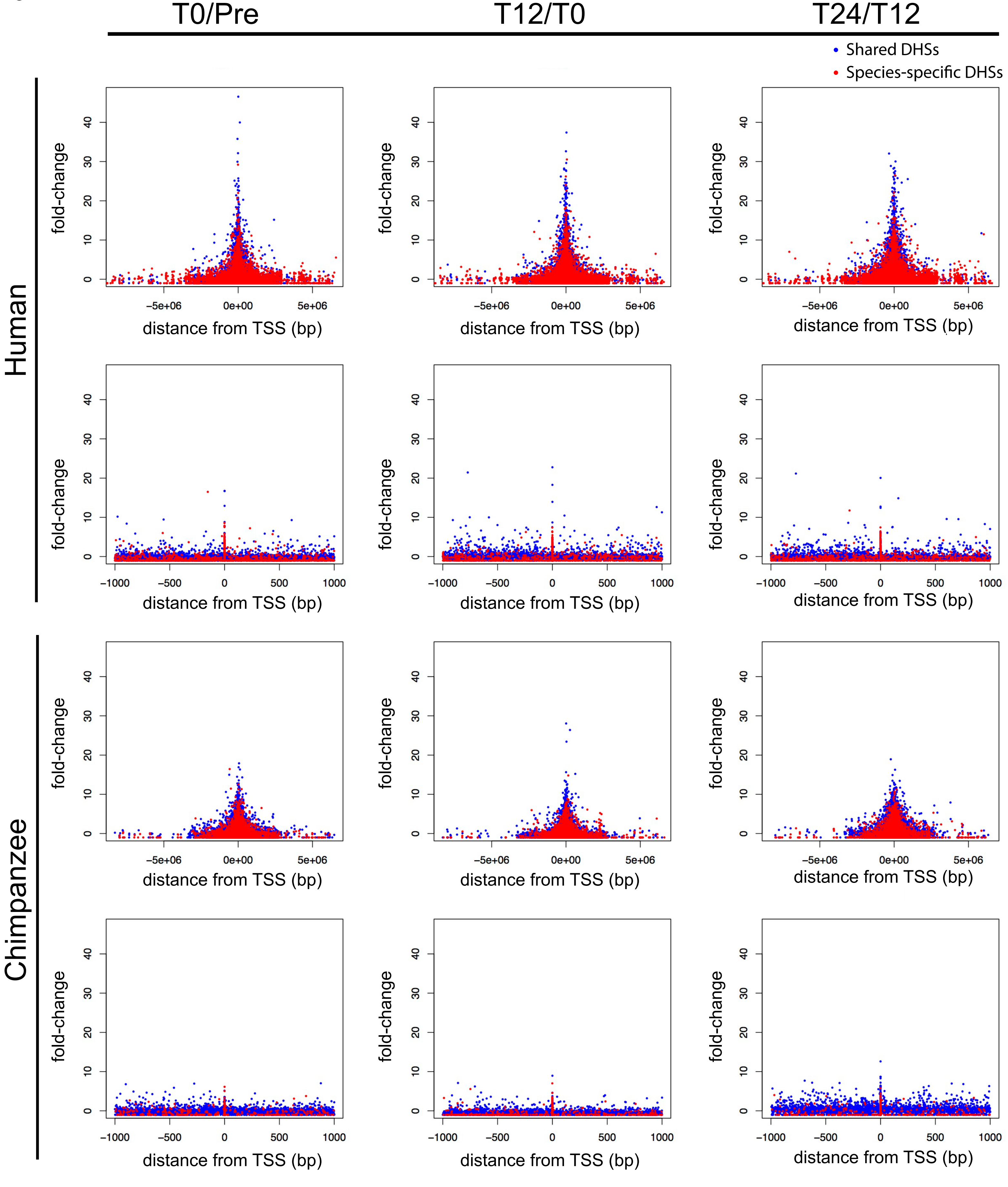

Figure S4. Changes in DHS activity increase with proximity to TSS. DHS distance from TSS versus fold-change in DHS activity between time points. Blue points are shared DHS sites, red points are species-specific DHS sites.

Figure S5

	Human			Chimpanzee				
Cluster Shape Pre, T0, T12, T24	Number of DHS sites	Number of Genes	Ratio DHS/ Gene	Number of DHS sites	Number of Genes	Ratio DHS/ Gene	DHS Ratio Human/ Chimp	Gene Ratio Human/ Chimp
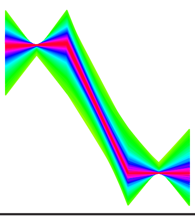	2674	2138	1.25	7329	4537	1.62	0.36	0.47
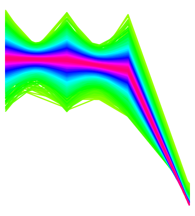	419	407	1.03	2534	2061	1.23	0.17	0.20
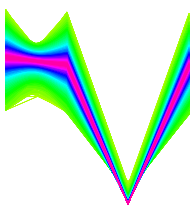	1369	1168	1.17	2370	1974	1.20	0.58	0.59
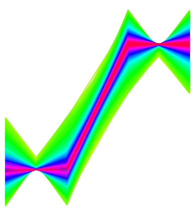	1776	1368	1.30	2370	0	0	n/a	n/a
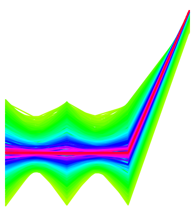	8452	4702	1.80	5848	3698	1.58	1.45	1.27
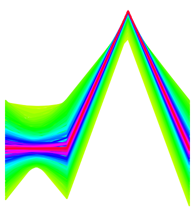	9746	5337	1.83	7434	4335	1.71	1.31	1.23
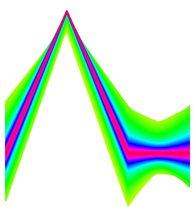	14330	6555	2.19	13557	6273	2.16	1.06	1.04
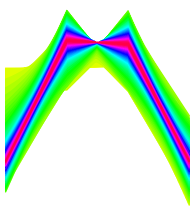	3139	2390	1.31	702	625	1.12	4.47	3.82
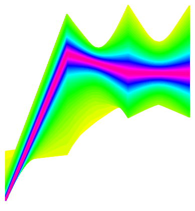	2966	2242	1.32	0	0	n/a	n/a	n/a
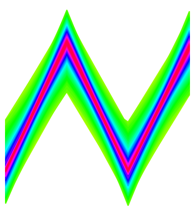	2986	2246	1.33	1231	1054	1.17	2.43	2.13
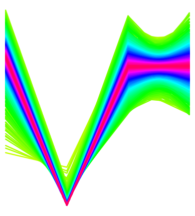	3585	2627	1.36	3768	2797	1.35	0.95	0.94
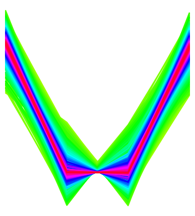	5870	3736	1.57	4820	3423	1.41	1.22	1.09
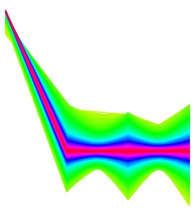	24065	8458	2.85	19451	7852	2.48	1.24	1.08
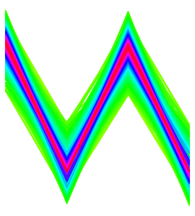	5021	3533	1.42	1445	1204	1.20	3.47	2.93
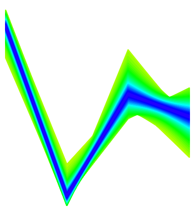	761	709	1.07	n/a	n/a	n/a	n/a	n/a
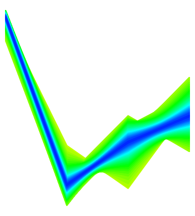	483	461	1.05	n/a	n/a	n/a	n/a	n/a
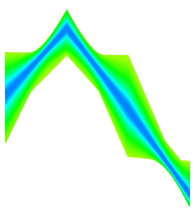	202	199	1.02	n/a	n/a	n/a	n/a	n/a
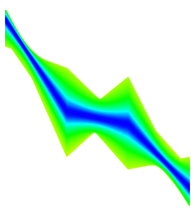	544	523	1.04	n/a	n/a	n/a	n/a	n/a
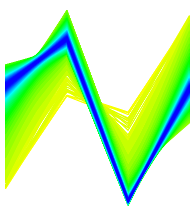	n/a	n/a	n/a	338	332	1.02	n/a	n/a
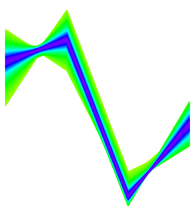	n/a	n/a	n/a	779	747	1.04	n/a	n/a

Figure S5. DHS sites and genes associated with clusters describing patterns of DHS activity over time. 20 cluster shapes that represent opening and closing chromatin through the time course of our assay in human and chimpanzee fibroblasts. The number of DHS sites that fit cluster shapes, and the number of genes associated with those DHS sites in both human and chimpanzee are described. Numbers of DHS sites per gene, and between-species ratios of DHS sites or genes that fit each cluster shape are calculated.

Figure S6

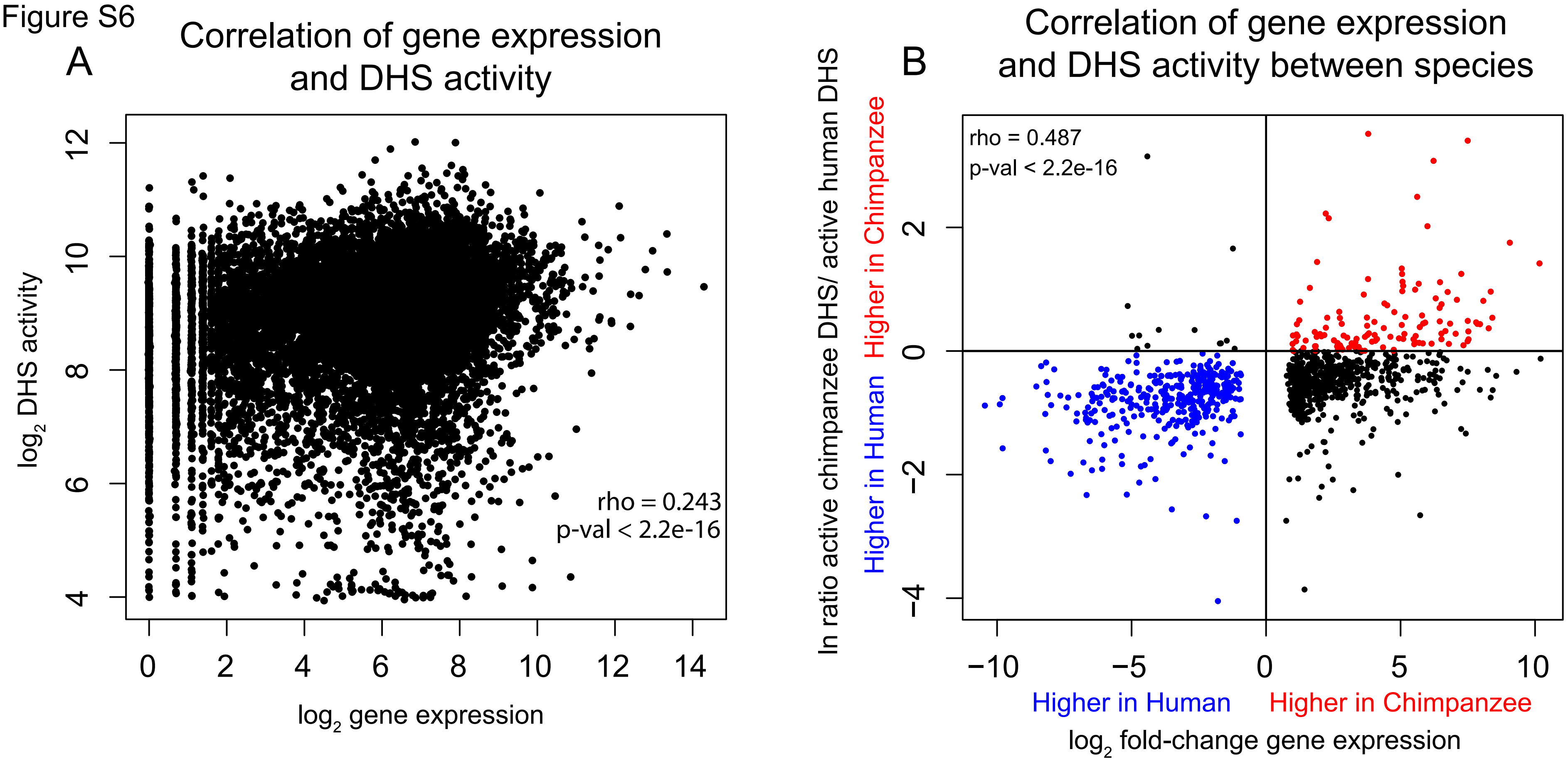

Figure S6. Correlations between gene expression and DHS activity. (a) The direct correlation of gene expression and DHS activity for one replicate at one time point (Human 1-pre) has a weak but positive correlation. (b) Comparison of $\log_2$ fold-change in gene expression between species across replicates versus the ratio of active DHS sites nearest each gene shows a moderate positive correlation.
