# Supplementary material for: Comparative Serum Challenges Show Divergent Patterns of Gene Expression and Open Chromatin in Human and Chimpanzee
Source: Genome Biol Evol. 2018 Mar 5;10(3):826–39. doi: 10.1093/gbe/evy041 (PMC5848805; doi:10.1093/gbe/evy041)
Supplement: Supplementary Data [file evy041_supp.zip › supplementary_text.doc]

**Supplementary Text**

**Human and chimpanzee DHS sites overlap with established open chromatin regions and contain established transcription factor binding motifs**

As a way to validate the DHS sites that we identified, we: 1) looked to see if the locations of our DHS sites have been identified in previous studies that used DNase-Seq, and 2) scanned for the presence of transcription factor binding motifs within our DHS sites. These comparisons act as a way to broadly characterize our DHS data set and suggest functional roles for these sites.

To see if the sites we identified in human and chimpanzee fibroblasts have been identified in other studies, we looked for overlap between our DHS sites and those reported in the Encyclopedia of DNA Elements (ENCODE) database (ENCODE Project Consortium 2012). Because some of our sites are only found in chimpanzee, we calculated the percentage of all sites (including shared, human-specific and chimpanzee-specific) that overlap with other data sets as well as the percentage of sites found in humans (including shared and human-specific). We compared our data to published DHS sites from fibroblasts and cancer cell lines and to human- and chimpanzee-specific DHS gains and losses in fibroblasts and lymphoblastoid cell lines (LCL) (Shibata et al. 2012) (supplementary table S2, Supplementary Material online).

In general, more of our sites overlap with those found in fibroblasts than cancer cell lines, except for one skin malignant melanoma cell line (supplementary fig. S3, Supplementary Material online). Although the percentages are comparable, about 4% to 6% more human DHS sites overlap with previously identified sites than the set of all human and chimpanzee sites. Between 48% and 69% of all sites, and 55% to 74% of human sites, overlap with previously identified DHS sites in fibroblasts. Between 25% and 52% of all our DHS sites, and 28% to 58% of human sites, overlap with those from cancer cell lines. To put the overlap with fibroblasts and cancer cell lines in context, we looked for overlap with unrelated tissues. DNase-Seq data from cerebellum, hepatocyte, and pancreas tissue have between 17% and 25% overlap with all of our DHS sites, and between 19% and 28% overlap with human DHS sites. In both cases, the cerebellum shows the highest percentage overlap and hepatocyte shows the lowest. Together these data show that chromatin activity of serum-challenged fibroblasts, based on overlap with published DHS data sets, is most similar to that seen in normal fibroblasts, and has more similarity with cancer cell lines than unrelated tissues, brain, liver, and pancreas.

Since DHS sites mark DNA regulatory regions, we looked for known transcription factor binding sites (TFBSs) that overlap with DHS sites in human and chimpanzee fibroblasts. We used the non-redundant set of transcription factor binding models from the JASPAR database (Mathelier et al. 2016) CORE collection of 386 transcription factor motifs to determine how many DHS sites from our study contain transcription factor binding motifs. 62% (163,603 of 264,091) of our DHS sites contain at least one TFBS (supplementary table S7, Supplementary Material online). Of shared DHS sites, 76% contain at least one TFBS, while the frequency is lower in human-specific (51%) and chimpanzee-specific (43%) sites. Within the shared DHS sites, there is an average of 3.01 TFBSs per DHS, and in species-specific DHS sites, there are 1.97 and 1.92 TFBSs in human and chimpanzee, respectively. While it seems that shared DHS sites have a higher concentration of binding sites, shared DHS sites, on average, are larger than species-specific DHS sites. The mean size of shared DHS sites that contain a binding site is 412 bp, whereas human- and chimpanzee-specific DHS sites are 187 and 176 bp, respectively. Thus, the TFBS frequency per kilobase is higher in species-specific sites than in shared sites even though these DHS sites overall contain fewer motifs.

**DHS sites in the genomic neighborhood of TSSs have the largest shifts in activity**

The majority of regulatory sites are distal to genes (ENCODE Project Consortium 2012). Thurman et al. (2012) report that 3% of DHS sites localize to transcription start sites (TSSs) and 5% are within 2.5 kb of a TSS. Of the DHS sites identified in our study, 3% of shared sites, 3.9% of human-specific sites, but only 1.6% of chimpanzee-specific sites localize to a TSS. Within 2.5 kb of the TSS are 8.5% of shared sites, 7.6% of human-specific sites, and 5.2% of chimpanzee-specific sites. Overall, shared sites are closer to TSSs than species-specific sites. The median distance to the nearest TSS is 51 kb, 66 kb, and 78 kb, for shared, human-specific, and chimpanzee-specific sites, respectively. As a proxy for DHS function, we calculated the mean -10log_10_ p-value associated with DHS sites among replicates and then measured the fold-change between time points. Shared sites and species-specific sites have greater fold-changes in activity over time with increasing proximity to the nearest TSS (supplementary fig. S4, Supplementary Material online). However, within 1 kb upstream of genic regions, there is not an increase in DHS change over time with increasing proximity to the TSS. At each time interval, DHS sites in human fibroblasts have higher maximal levels of change and more sites undergoing changes in activity.
